# Ten simple rules for designing and running a computing minor for bio/chem students

**DOI:** 10.1371/journal.pcbi.1010202

**Published:** 2022-07-14

**Authors:** Rochelle-Jan Reyes, Nina Hosmane, Shasta Ihorn, Milo Johnson, Anagha Kulkarni, Jennifer Nelson, Michael Savvides, Duc Ta, Ilmi Yoon, Pleuni S. Pennings

**Affiliations:** 1 Department of Biology, San Francisco State University, San Francisco, California, United States of America; 2 Department of Computer Science, San Francisco State University, San Francisco, California, United States of America; 3 Herbert Wertheim School of Public Health and Human Longevity Science, UC San Diego, San Diego, California, United States of America; 4 Department of Organismic and Evolutionary Biology, Harvard University, Cambridge, Massachusetts, United States of America; 5 Department of Integrative Biology, University of California, Berkeley, California, United States of America; Carnegie Mellon University, UNITED STATES

## Abstract

Science students increasingly need programming and data science skills to be competitive in the modern workforce. However, at our university (San Francisco State University), until recently, almost no biology, biochemistry, and chemistry students (from here bio/chem students) completed a minor in computer science. To change this, a new minor in computing applications, which is informally known as the Promoting Inclusivity in Computing (PINC) minor, was established in 2016. Here, we present the lessons we learned from our experience in a set of 10 rules. The first 3 rules focus on setting up the program so that it interests students in biology, chemistry, and biochemistry. Rules 4 through 8 focus on how the classes of the program are taught to make them interesting for our students and to provide the students with the support they need. The last 2 rules are about what happens “behind the scenes” of running a program with many people from several departments involved.

## Introduction

Science students increasingly need programming and data science skills to be competitive in the modern workforce [1–3]. This is clearly true for those who are interested in research or a career in the biotechnology industry, but we would argue that pre-health students, those who are interested in becoming teachers, and most others could also benefit from a basic knowledge of programming and data science.

At our university (San Francisco State University), until recently, almost no biology, biochemistry, and chemistry students (from here bio/chem students) completed a minor in computer science. A group of faculty decided to try and change this by creating a new minor in computing applications, which is informally known as the Promoting Inclusivity in Computing (PINC) minor. Our goal with this initiative was to design a new minor in computer science that would be accessible and appealing to bio/chem students.

The minor was designed for undergraduate students in biology, biochemistry, and chemistry, to obtain over the course of 2 years with 15 units of computer science courses. The minor has 3 main learning objectives for students interested in obtaining the minor: (1) demonstrate foundational knowledge of computational skills and their application to multidisciplinary problems; (2) demonstrate functional knowledge of machine learning concepts, tools, and techniques; and (3) demonstrate ability to synthesize computational and machine learning skills around the big questions in diverse fields (e.g., biotechnology) to develop solutions to real-life problems. The courses provide students an introduction to computing skills with an interdisciplinary approach on how these skills can be applied to the life and health sciences.

**Fig 1:**
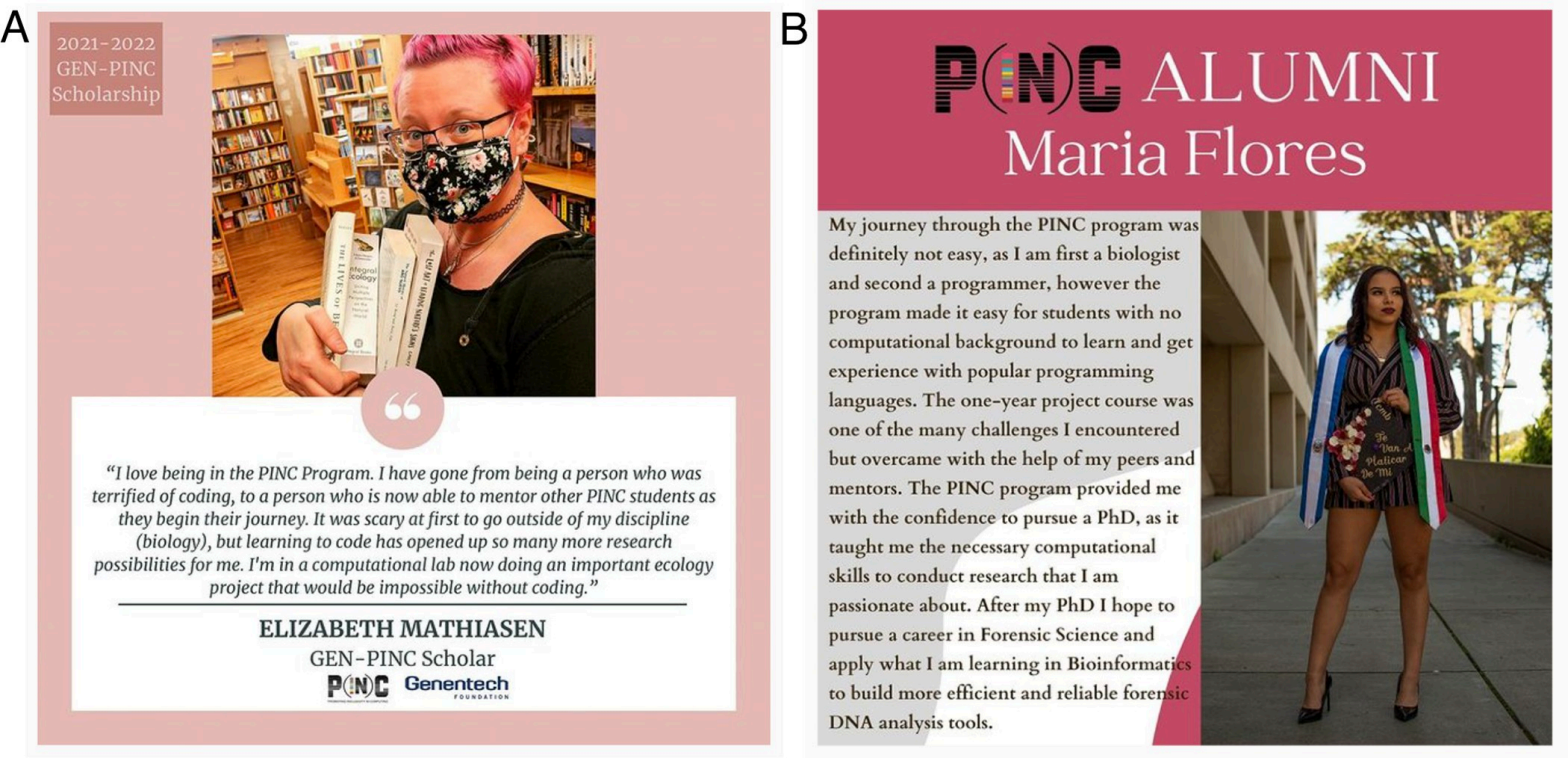
Two images from the PINC Instagram feed (https://www.instagram.com/sfsupinc/), where we celebrate current students and alums. On the left, Elizabeth Mathiasen (current PINC student) says: *“I love being in the PINC Program*. *I have gone from being a person who was terrified of coding*, *to a person who is now able to mentor other PINC students as they begin their journey*. *It was scary at first to go outside of my discipline (biology)*, *but learning to code has opened up so many more research possibilities for me*. *I’m in a computational lab now doing an important ecology project that would be impossible without coding*.” On the right, former PINC student and current UCLA PhD student Maria Flores says: *“My journey through the PINC program was definitely not easy*, *as I am first a biologist and second a programmer*, *however*, *the program made it easy for students with no computational background to learn and get experience with popular programming languages*. *The one-year project course was one of the many challenges I encountered but overcame with the help of my peers and mentors*. *The PINC program provided me with the confidence to pursue a PhD*, *as it taught me the necessary computational skills to conduct research that I am passionate about*. *After my PhD*, *I hope to pursue a career in Forensic Science and apply what I am learning in Bioinformatics to build more efficient and reliable forensic DNA analysis tools*”.

Since 2016, 4 cohorts of undergraduate students in biology, chemistry, and biochemistry have completed the requisites necessary to obtain the minor. Many of our PINC minor alums have gone on to jobs in the industry, PhD programs, and medical school, among other careers (Fig 1). We are proud of the program we have created, and even more proud of the students we work with.

Through time, we have found several ways to improve the program. Here, we share the lessons we learned in the form of 10 rules for designing a computing minor for non-CS students. We believe that at many institutions for higher education, computing talent is currently untapped, and we hope that our experience can help you set up your own program, so that more bio/chem students can learn coding and data science skills! (https://pinc.sfsu.edu/pinc)

### A. Setting up the program and recruiting students

#### RULE 1: Narrow your target audience

The courses of the new computing applications minor (PINC minor) were initially open to all majors, but we quickly learned that a narrower audience would work better for our program. We now offer courses specifically designed for bio/chem students, and we use in-class examples and projects focused on topics that are of interest to these students, such as sequence analysis. This has made it easier to show students how computing skills can be useful in their field of interest, and the familiar context of biology and (bio)chemistry helps students learn foundational CS concepts. For example, students learn about loops by looking at nucleotides or codons in a DNA sequence.

Another benefit of a narrow focus is that the shared identity of being a bio/chem student helps students feel a sense of belonging in the PINC program and creates a less intimidating and safer classroom environment. We have biology or chemistry faculty teach the first course in the program, which helps transition a cohort of students with a shared interest in biology or (bio) chemistry to CS content. Finally, because of the focus of our program, it has been possible to make meaningful connections with local biotech companies who are interested in connecting with students with computing and science skills.

#### RULE 2: Make the program welcoming for students with no prior coding experience

Due to inequitable access to computer science education prior to college, many students feel unprepared for introductory CS coursework [4]. In conversations with students at SFSU, many indicated that they avoided taking CS classes because they felt like they were already behind their peers at the start of a difficult course [5,6]. When students do sign up for a CS course, feelings of imposter syndrome and the experience of stereotype threat can also contribute to attrition [5,7].

**Fig 2:**
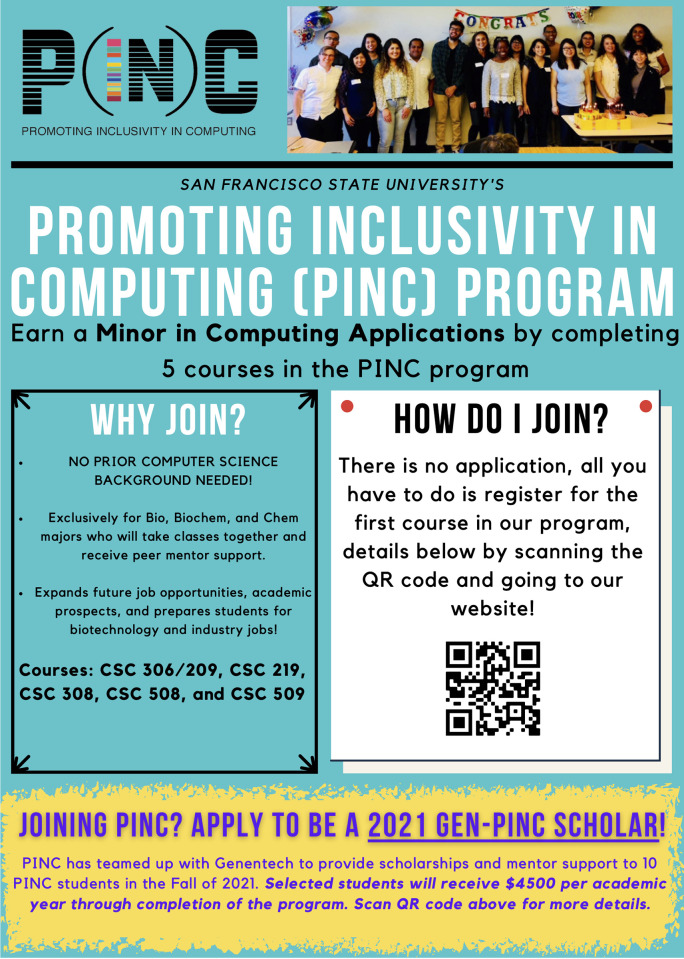
A flyer to promote the PINC program to bio/chem undergraduate students.

When we recruit for our program, we acknowledge students’ concerns and demonstrate the inclusivity of our program through posters, emails, class visits, informational sessions, a website, and social media posts (Fig 2). In all promotional materials, we emphasize that there is no computer science experience required. Students join the program by simply signing up for the first class. We ensure any student who needs a laptop can get one for free through a loan program. In our introductory class, we start slow because we know that there are many students who have never coded before. We also incorporate lectures on stereotype threat, imposter syndrome, and growth mindset in the courses to address psychological barriers directly and to create an environment where these issues are openly discussed by students and faculty. By removing or reducing practical and psychological roadblocks, we open the door of the introductory CS courses for many students who previously were excluded.

#### RULE 3: Make sure there is a tangible and achievable incentive for the students, such as a minor or certificate

Students have many different sources of motivation, but we believe that the possibility of obtaining a minor or certificate can provide a strong incentive for students to stay in the program even when it is difficult. Our students earn a minor in “Computing Applications” when they finish all 5 courses. From 2021, our students can also earn a certificate in “Machine Learning and Data Science for Biotechnology” by taking 3 additional units of coursework.

**Fig 3:**
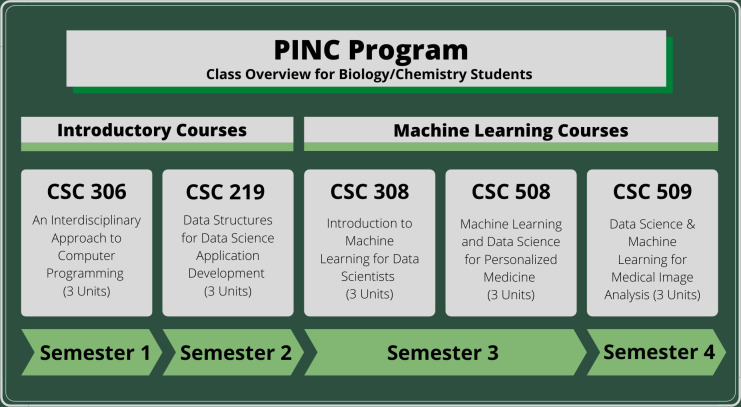
The curricular structure of the PINC program as offered in 2021–2022. A full list of classes is included as a supplement S1 Text.

When we designed the PINC program, we wanted to make sure that the minor was both useful and achievable. A previously existing CS minor consisted of 7 CS classes that had no link to biology or (bio)chemistry. Between 2003 and 2017, only a single biology student ever graduated with that CS minor. The new minor has only 5 classes, which we hoped would allow more students to finish (Fig 3). All 5 classes are newly created for the PINC program. In 2018, the first cohort of students finished the minor. Between 2018 and 2021, 22 bio/chem students completed all courses and graduated with a minor in “Computing Applications.” This number, however, doesn’t reflect the actual number of students who benefited from the PINC program. Some students finished all 5 courses, but haven’t finished their major degree yet, thus are not counted as having graduated with a minor.

In addition, many of our students are not in it for the minor. Some students finished all their courses but they aren’t eligible for the minor, for example, if they are MS students. Other students take one or several PINC courses out of interest, but never intend to do the entire minor. Thus, we generally see a larger number of students taking introductory PINC courses. The first class of the PINC program, CSC 306, has now been taken by 186 undergraduate students and 55 MS students.

### B. Supporting the students while they’re in the program

#### RULE 4: Include mandatory meetings with trained peer mentors

From the beginning of the PINC program, we have hired students as (peer) mentors to support the students in the PINC classes. These peer mentors are current and former PINC students as well as CS students. For each course, students are grouped in teams of 4 to 5 students, and each team is assigned a mentor. The team meets with the mentor once a week, and each mentor supports 2 student teams. Our mentor program is different from typical TA support in classes, because all students are automatically assigned a mentor and attendance in the mentor meetings counts for 10% of the course grade. By incentivizing students with a grade, they are likely to commit to regular mentor meetings early in the semester, which helps them build connections they can rely on for support later in the semester. We believe that our mentors are one of the reasons why students stay in the PINC program. During program and course evaluations, the students often mention how much they benefit from the mentors. For example, one student said: “My mentor is very good at going over the notebooks and projects. They explain concepts well and break them down when we are struggling with something.” Another student said: “It felt like a student-to-student relationship with my mentor. That made communicating and working with my mentor very comfortable for me.”

**Fig 4:**
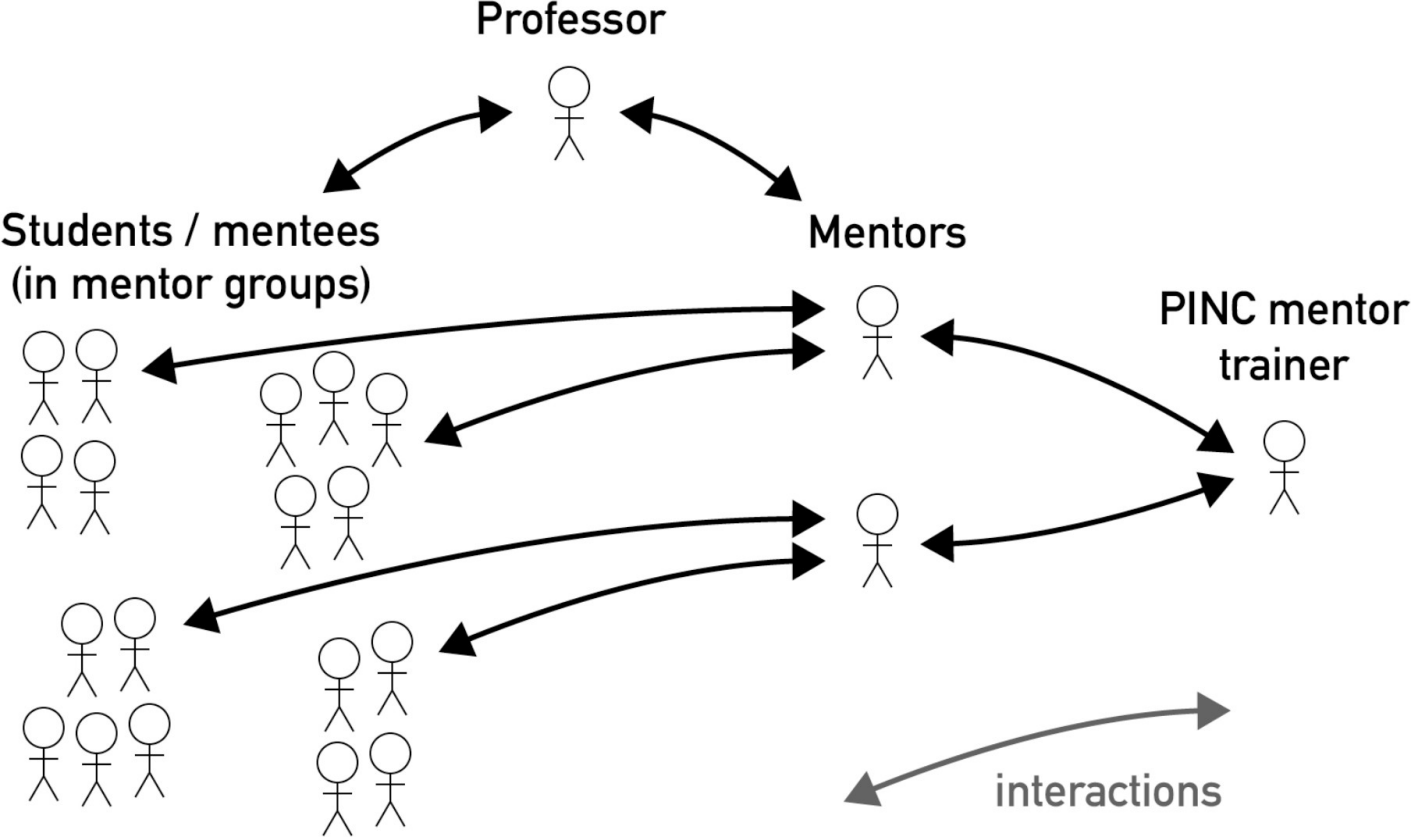
Important players in the PINC mentor program: professor, mentees, mentor, and mentor trainer.

To make sure the mentors in the PINC program are effective in their work, we have developed a monthly mentor training curriculum (Fig 4). Here, the mentors learn how they can create good relationships between themselves and their mentees (e.g., by setting up one-on-one meetings a few times in the semester) and how they can encourage good relationships among their mentees as well. Evaluations show that the mentors also benefit from this training program. For example, 1 mentor reported: “It helped me identify my strong points, boost my confidence, and landed me a job as an instructor!” Another mentor said: “I feel that it [being a PINC mentor] has given me the ability to lead a small group.”

#### RULE 5: Take concrete steps to foster a sense of community

Students learn better when they feel part of a learning community [8], but simply telling students “you’re part of our community” isn’t sufficient to create an environment where they feel an authentic connection with their peers and faculty [9,10]. This is particularly true in the face of the challenges inherent in remote learning, which has become an integral part of the PINC program in the wake of the COVID-19 pandemic. We take concrete steps to foster a strong sense of community between students, staff, and faculty, and relationship building has become a core part of our efforts to continually improve the program.

PINC has a cohort-based structure, and coursework relies heavily on project-based, small-group learning, which creates many opportunities for meaningful peer interaction. PINC’s peer mentoring program and online communication channels (e.g., Slack, Discord) create systems for students to seek and provide support outside of class. PINC faculty plan events (both in-person and virtually) to celebrate student successes, showcase student work, and provide professional networking opportunities; these events include formal and informal opportunities for faculty, staff, students, and industry partners to share a meal, watch presenters and speakers, and engage in discussion.

The PINC program has an active presence on social media and maintains mailing lists; through both of these avenues, PINC communicates frequent program updates, including career and professional development resources, student highlights, and PINC-specific internship opportunities. All of these systems contribute to a sense of community within the program, creating a learning environment where—as a student noted during an evaluation interview—“We are a little PINC family, we know each other, see each other in the hall.”

#### RULE 6: Make inclusivity a part of the curriculum

**Fig 5:**
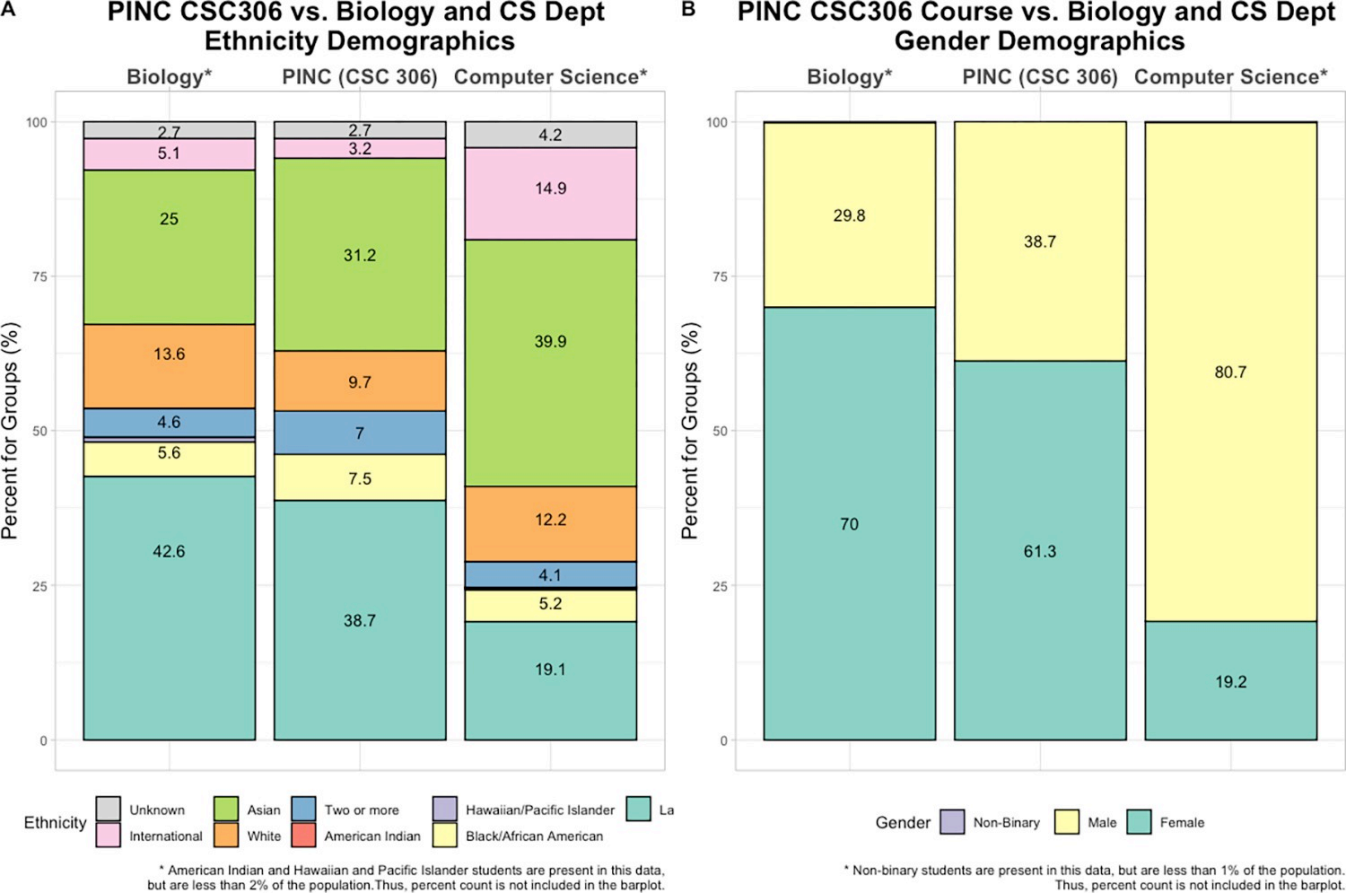
(**A**) Demographics of biology major (left), students in CSC 306 (the first class of the PINC minor, middle) and CS majors (right). Approximately 48% of biology majors are black or Latinx, compared to 24% in the CS major. The students in the PINC class CSC 306 reflect the demographics of the biology program; 46% of them are black or Latinx. Data from SFSU Institutional Research, based on 186 undergraduate students who took CSC 306 between 2016 and 2021. *American Indian and Hawaiian and Pacific Islander students are present in this data but are less than 2% of the population. Thus, they are present within the bar plot but the numerical label is not present in the figure. (**B**) Demographics of biology major (left), students in CSC 306 (the first class of the PINC minor, middle) and CS majors (right). Approximately 70% of biology majors identify as women, compared to 19.2% in the CS major. Approximately 61.3% of the students in the PINC class CSC 306 identify as women. Data from SFSU Institutional Research, based on 186 undergraduate students who took CSC 306 between 2016 and 2021. *Nonbinary students are present in this data, but are less than 1% of the population. Thus, they are present within the bar plot but the numerical label is not present in the figure. In addition, students who identify as nonbinary may not be listed as such in the university data base.

On our campus, and elsewhere, computer science courses tend to attract more men and more white and Asian students, relative to the entire campus population. In biology, biochemistry, and chemistry, we find relatively more women and more Latinx and black students, compared to computer science (Fig 5A and 5B). One of our goals for the PINC program is to train women and nonbinary students and more students from underrepresented groups.

In our program, we try to recruit a diverse group of students each year. To do this, we make sure that our recruitment materials (flyers, website, and slides) have welcoming text and images of the diverse students who are already in the program. We also make an effort to highlight diverse scientists in the classroom, for example, by working with “scientist spotlights” [11], by including work by diverse scientists in the class curriculum, and by assigning reading materials about or by diverse scientists. Now that we are in the fifth year of the program, our alums also play a role, as we can show in the classroom and on social media how women, nonbinary people, and people of color have been successful in the PINC program. We also make an effort to hire a diverse team of teachers, mentors, and staff.

One way we try to gauge whether our efforts in inclusivity are successful is by comparing the demography of the PINC students with the demography of the biology department from which we recruit the majority of our students. If our program is welcoming to all students, we’d expect the demographics of these 2 groups to be similar. We find indeed that the undergraduate students who take CSC 306 (the first class of the PINC minor) have similar demographics when compared to biology students at SFSU. Approximately 46% of them are black or Latinx and 61% of them identify as women (Fig 5A and 5B). On the other hand, students in the computer science department are less likely to be black or Latinx and to identify as women, which shows that efforts like PINC are needed.

#### RULE 7: Be compassionate and flexible

We want students to be able to succeed in our program even when they face difficulties inside and outside of class. Because our students are taking challenging CS classes in addition to their regular classes in biology, chemistry, and biochemistry, and because many of them have jobs or care for family members, they can fall behind on coursework. We try always to approach the students with compassion and flexibility [12]. When a student is dealing with difficulties in their life, we believe what they say, and we offer help in the form of extra one-on-one office hours and extensions for assignment due dates. As a faculty group, we routinely talk (see Rule 9) about students who are struggling, and brainstorm together how we can help them get back on track.

Some concrete steps we have taken to prevent students from falling behind are the following. First, we offer the option of taking the first 2 courses in the program on a Credit/No-Credit basis (i.e., Pass/Fail) to reduce grade-based stress among students. Second, we are generally open to extensions of deadlines and accept late work or corrections for partial or full credit. Third, we design our curriculums with our students in mind, knowing that they have other classes and busy lives, and try not to pack too much content in each class. Fourth, we encourage students to take a class a second time if they feel like they could benefit from it. And finally, we offer multiple avenues for students to voice concerns and to ask for help (e.g., faculty, mentors, program support staff).

#### RULE 8: Provide professional development opportunities for students

As part of our program, we offer professional development opportunities for students and mentors to help them prepare for the next steps in their career, whether it is graduate school, industry, medical school, etc. We want our students to know what is possible for them and to be prepared to apply for opportunities and then succeed in them. Examples of professional development opportunities we have offered include CV and resume workshops, scholarship and grant writing workshops, information sessions with local industry partners, and presentations by PINC alums. We also keep track of relevant opportunities such as jobs and internships and share these with our students.

Some of the professional development activities are offered as part of the classes, while in other cases, they are offered outside of the classroom. We communicate these opportunities to our students through multiple channels such as in class, through email distribution lists, newsletters, social media, and through a career portal on our website. We form and maintain relationships with local industry partners in the biotechnology and pharmaceutical space and have even collaborated with industry to create paid internship opportunities that are specifically designed for the students in our program.

### C. Behind the scenes

#### RULE 9: Make sure there is ample communication in the team of faculty and staff

**Fig 6:**
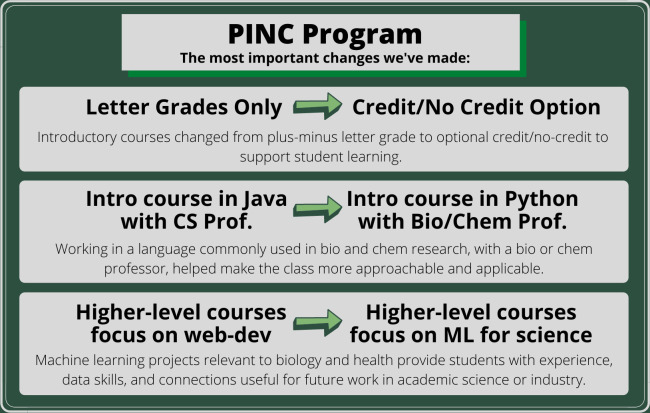
List of the main changes we made to the PINC minor between 2016 and 2021.

Successful management of the PINC program requires frequent communication and collaboration between several stakeholders, including faculty, staff, administrators, evaluators, mentors, and students. While the Computer Science department coordinates the program, our stakeholders come from several different fields (e.g., computer science, biology, chemistry, and psychology). This provides a unique opportunity for the program to take an interdisciplinary approach to computational learning for students, but also comes with the challenges inherent in communicating across disciplines [13]. The PINC team has several groups that meet regularly, including one for program faculty, staff, and evaluators. This team meets every other week and common discussion topics include: troubleshooting programmatic issues that arise; providing support for teaching faculty in developing and implementing effective teaching strategies; checking in about students who are struggling, sharing success stories of our students; and discussing evaluation, grant-writing, and research efforts. In addition to creating space for eye-opening interdisciplinary communication and perspective-sharing, these meetings are also an integral part of the program’s flexibility. Over the years, the program has shifted significantly (we learned early on that the first version isn’t always perfect!), and these meetings have been a key part of allowing the program to adapt quickly to challenges and to collaboratively solve any issues that arise. In Fig 6, we list some of the most important changes we made in the program between its inception in 2016 and 2021.

#### RULE 10: Get funding to hire staff, support faculty, and organize events

We feel that generous monetary support from government grants, philanthropic institutions, and private industry partners have been extremely helpful in starting and running the PINC minor. The PINC program began with a grant from the Center for Advancing Women in Technology (CAWIT), a 501(c)(3). After that, we were able to acquire an NSF IUSE grant, and most recently we partnered with Genentech and Genentech Foundation. The funds from CAWIT and NSF allowed the PINC program to develop and experiment with classes, to hire a mentor trainer, and to hire (peer) mentors. The funds from the Genentech Foundation allowed our program to expand services to students, including a limited number of scholarships for PINC students.

One of the main reasons the PINC program is successful is because of a well-developed peer-mentoring structure, but this component costs money as we pay the mentors and the mentor trainer. On the back-end, having staff who can organize external relations and program communications has proven essential to the growth and continued success of the program. Funding has also been essential for supporting faculty—for example, to reduce someone’s teaching load when they need time to develop a new class. In the first years of the PINC program, we also used external grants to pay lecturers, when enrollment in the PINC classes was low and without these funds, the college would have canceled the “under enrolled” classes.

Funding is only one aspect of support. We are also supported by many colleagues on campus. Relationships with like-minded STEM education communities strengthens our program. The PINC program has close ties with several other programs and clubs on campus who hold similar values around equity and diversity in STEM, and we regularly share information on grants, internships, and classes.

## Conclusions

To summarize, we believe that it is important to align computing programs with students’ interest, motivation, and current knowledge. Any computing program should consider the fact that students may be reluctant and uncertain about their own talent—so community, purpose, and compassion are important to foster interest in the fields and confidence in oneself. Finally, the people who are running the program need to form a community as well, to support each other and the students, as well as to be adaptable throughout the process based on what the students and the program need.

Many students are interested in computing, and once they try it in a supportive environment, they discover talent they didn’t know they had. Institutions of higher education need to offer programs that work for their students. If you decide to embark on this journey, we hope that you’ll find it as rewarding as we have found it to be.

## Supporting information

S1 Text List of classes in the PINC program (Minor in computing applications) as of Spring 2022. (PDF)
