# Supplementary material for: Ten simple rules for designing and running a computing minor for bio/chem students
Source: PLoS Comput Biol. 2022 Jul 14;18(7):e1010202. doi: 10.1371/journal.pcbi.1010202 (PMC9282537; doi:10.1371/journal.pcbi.1010202)
Supplement: S1 Text — (PDF) [file pcbi.1010202.s001.pdf]

# Appendix

List of classes for the PINC program / Minor in Computing Applications at San Francisco State University as of Spring 2022, with bulletin descriptions

## CSC 306 An Interdisciplinary Approach to Computer Programming (Units: 3)

Prerequisites: Restricted to Biology, Chemistry, and Biochemistry majors and pre-majors; upper-division standing; or permission of the instructor.

Basics of programming for interdisciplinary problem-solving, using Python. Topics include basic building blocks of programming (variables, control statements, loops, function, abstraction, and more) and problem-solving approaches relevant to problems in the natural sciences.

## CSC 219 Data Structures for Data Science Application Development (Units: 3)

Prerequisite: CSC 210 or equivalent.

Focuses on learning about and utilizing data structures and algorithms effectively for developing data science applications. Utilizes Python and Jupyter Notebook.

## CSC 308 Introduction to Machine Learning for Interdisciplinary Data Scientists (Units: 3)

Prerequisite: CSC 219.

Introduction to the basic machine learning concepts and tools. Focus on applying them for application development, linear model, deep neural network and transfer learning using Python, Tensorflow, and Keras. (Plus-minus letter grade only)

## CSC 508 Machine Learning and Data Science for Personalized Medicine (Units: 3)

Prerequisite: CSC 308 (may be taken concurrently).

Exploration of the concepts and tools needed to analyze, interpret, and reason from genomic datasets to help medical professionals better treat their patients. Diseases often affect patients differently depending on their genetic background. To make personalized medicine a reality, it is necessary to leverage the data from genomic datasets. Genomic data science applies machine learning and data science to the genome to better diagnose and treat patients. (Plus-minus letter

**grade only)**

## CSC 509 Data Science and Machine Learning for Medical Image Analysis (Units: 3)

Prerequisite: CSC 308 (may be taken concurrently).

Exploration of the application of state-of-the-art deep learning models to medical image analysis: the task of identifying objects such as tissue, bone within an image. Discussion of topics central to deep learning-based image analysis, and practical application of these concepts in three hands-on case studies. (Plus-minus letter grade only)
